# Stochastic Memristive Interface for Neural Signal Processing

**DOI:** 10.3390/s21165587

**Published:** 2021-08-19

**Authors:** Svetlana A. Gerasimova, Alexey I. Belov, Dmitry S. Korolev, Davud V. Guseinov, Albina V. Lebedeva, Maria N. Koryazhkina, Alexey N. Mikhaylov, Victor B. Kazantsev, Alexander N. Pisarchik

**Affiliations:** 1 Institute of Biology and Biomedicine, National Research Lobachevsky State University of Nizhny Novgorod, 603950 Nizhny Novgorod, Russia; gerasimova@neuro.nnov.ru (S.A.G.); lebedeva@neuro.nnov.ru (A.V.L.); kazantsev@neuro.nnov.ru (V.B.K.); 2 Research Institute and Technology, National Research Lobachevsky State University of Nizhny Novgorod, 603950 Nizhny Novgorod, Russia; belov@nifti.unn.ru (A.I.B.); dmkorolev@phys.unn.ru (D.S.K.); guseinov@phys.unn.ru (D.V.G.); mian@nifti.unn.ru (A.N.M.); 3 Research and Educational Center “Physics of Solid State Nanostructures”, National Research Lobachevsky State University of Nizhny Novgorod, 603950 Nizhny Novgorod, Russia; mahavenok@mail.ru; 4 Laboratory of Neuroscience and Cognitive Technology, Innopolis University, 420500 Innopolis, Russia; 5 Center for Neurotechnology and Machine Learning, Immanuel Kant Baltic Federal University, 236016 Kaliningrad, Russia; 6 Center for Biomedical Technology, Universidad Politécnica de Madrid, Pozuelo de Alarcón, 28223 Madrid, Spain

**Keywords:** memristive device, neuron-like oscillator, stochastic dynamics, synchronization, neuromorphic circuit, FitzHugh–Nagumo neuron

## Abstract

We propose a memristive interface consisting of two FitzHugh–Nagumo electronic neurons connected via a metal–oxide (Au/Zr/ZrO_2_(Y)/TiN/Ti) memristive synaptic device. We create a hardware–software complex based on a commercial data acquisition system, which records a signal generated by a presynaptic electronic neuron and transmits it to a postsynaptic neuron through the memristive device. We demonstrate, numerically and experimentally, complex dynamics, including chaos and different types of neural synchronization. The main advantages of our system over similar devices are its simplicity and real-time performance. A change in the amplitude of the presynaptic neurogenerator leads to the potentiation of the memristive device due to the self-tuning of its parameters. This provides an adaptive modulation of the postsynaptic neuron output. The developed memristive interface, due to its stochastic nature, simulates a real synaptic connection, which is very promising for neuroprosthetic applications.

## 1. Introduction

The design of compact neuromorphic systems, including micro- and nanochips, capable of reproducing information and computational functions of brain cells is a great challenge of modern science and technology. Such systems are of interest for both fundamental research in the field of nonlinear dynamics and the synchronization of complex systems [1,2,3,4,5,6,7], as well as medical applications in the devices for monitoring and stimulating brain activity in the framework of neuroprosthetic tasks [8,9,10]. Due to their importance, memristive devices have recently become the subject of intense research, especially in the area of neuromorphic and neurohybrid applications [11,12,13,14,15,16,17]. Neuromorphic technologies are especially relevant for intelligent adaptive automatic control systems—biorobots. It is also worth noting that the construction and creation of electronic neurons and synapses (connections between neurons) based on thin-film memristive nanostructures is a fast-growing area of interdisciplinary research in the development of neuromorphic systems [18,19,20].

The history of neuromorphic technologies began in the late 1980s with the emergence of computation machines, and since then, significant advances have been achieved in electronics, physics of micro- and nanostructures, and solid-state nanoelectronics. The careful development of neuron-like electrical circuits made it possible to reproduce basic neural behaviors, such as resting, spiking, and bursting dynamics, as well as more sophisticated regimes, including chaos and multistability [21,22,23,24,25].

A memristive device is usually based on the Chua’s model [19], which is an element of an electrical circuit capable of changing resistance depending on an electrical signal entering its input. In recent decades, various thin-film memristive nanostructures have been created. They are capable of changing their conductivity under the action of a pulsed signal [26,27], which makes the memristor an almost ideal electronic analogue of a synapse [13]. A synapse is known to be a communication channel between neurons that provides unidirectional signal transmission from a transmitting (presynaptic) neuron to a receiving (postsynaptic) neuron. This communication channel ensures the propagation of a nerve impulse along the axon of the transmitting cell.

The synaptic communication results in synchronization of postsynaptic and presynaptic neurons. Neural synchronization was extensively studied using various mathematical models and described in terms of periodic solutions [3,6,28,29,30,31,32,33,34,35]. Such artificial synapses were implemented as electronic circuits that convert pulses of presynaptic voltage into postsynaptic currents with some synaptic amplification. Different strategies were used for the hardware implementation of synaptic circuits, e.g., an optical interface between electronic neurons [4,5,7].

Recent advances in nanotechnology allowed for miniaturization of artificial synapses by creating memristive nanostructures that mimic dynamics of real synapses. Among various candidates for the role of electronic synapses, memristive devices have a great potential for implementing massive parallelism and three-dimensional integration in order to achieve good efficiency per unit volume [36,37,38]. In this regard, it is important to create a memristor-based neuromorphic system capable of processing neuron-like signals.

Recently, the interaction between electronic neurons through a metal-oxide memristive device was successfully implemented in hardware [39]. The prerequisite for such a device was the study of the interaction of Van der Pol generators via a memristor [40]. Later, a significant effort was invested in theoretical research to study synchronization between neuron-like generators connected through a memristive device [14,41]. However, to the best of our knowledge, experimental studies of the dynamics of FitzHugh–Nagumo (FHN) neurons connected by a memristive synapse have not yet been carried out. We believe that the creation of neuromorphic memristive systems will lead to the production of simple and compact neuroelements based on memristive devices capable of imitating the electrophysiological behavior of real neurons.

At the same time, a memristive device made of metal oxides is of interest not only for experimental research, but also for theoretical studies. Neuromemristive models were found to exhibit complex dynamics, including chaos and chimeras [42,43], the study of which can contribute to the fundamental theory. On the other hand, many theoretical “memristive” neural models reported in the literature have nothing to do with the concept of memristive elements [44]. Therefore, the development of adequate mathematical models that can simulate real laboratory neuromemristive experiments is an actual problem.

Summarizing all the above, significant theoretical investigations of memristors and the possibility of their use as a part of neuromorphic systems were performed. In particular, not only dynamical were effects simulated, but also the simplest learning rules were implemented [45,46,47,48,49,50,51,52]. Currently, technologies are being developed to improve the characteristics of memristive devices in order to create reliable memristive networks capable of solving some mathematical tasks [53], classifying images [54,55,56,57,58], etc. [59,60,61]. Despite impressive theoretical results in the development of neuromorphic memristive systems, the experimental research of laboratory memristive devices, rather than their substitutes based on transistors or resistors as parts of dynamical systems, was not carried out because of high complexity of this task, which requires the cooperation of nanotechnologists, physicists, and neuroscientists.

In this work, we experimentally implement a memristive interface based on the metal–oxide nanostructure that acts as a synaptic interface connecting two electronic FHN neural generators. The interface allows for the analog simulation of the adaptive behavior and neural timing effects, which can be associated with synaptic plasticity. We also investigate the stochastic properties of the memristive device. For the first time, to the best of our knowledge, we perform an experimental study on such a memristive neural system and compare experimental results with numerical simulations.

## 2. Materials and Methods

In order to simulate neural dynamics, we explored two FHN neuron generators with cubic nonlinearity constructed using diodes [7,22]. The dynamics of the presynaptic FHN neuron was modeled by the normalized equations obtained with the Kirchhoff law [21] as follows:

(1) $$\begin{array}{l} \frac{du_{1}}{dt}=f(u_{1})-v_{1}\\ \frac{dv_{1}}{dt}=\varepsilon (g(u_{1})-v_{1})-I_{1} \end{array}$$

where *u*_1_ is the membrane potential of the presynaptic neuron, *ν*_1_ is the “recovery” variable related to the ion current, *f*(*u*_1_) = *u*_1_−*u*_1_^3^/3 is the cubic nonlinearity, *I*_1_ is the depolarization parameter characterizing the excitation threshold, and ε is a small coefficient. If *u*_1_ < 0, the function *g*(*u*_1_) = *αu*_1_, and if *u*_1_ ≥ 0, *g*(*u*_1_) = *βu*_1_ (*α*, *β* being the parameters that control, respectively, the shape and location of the *ν*-nullcline [22]).

The memristive device model was developed based on a standard approach to reflect the dynamical response of a memristor to electrical stimulation. The model describes a change in resistance, similar to potentiation and depression, based on physical laws identified in experiments [62]. The memristor model is given by the complex function:

(2) $$\begin{array}{l} j=wj_{lin}+(1-w)j_{nonlin}\\ j_{lin}=u_{1}/\rho \\ j_{nonlin}=u_{1}\exp(b\sqrt{u_{1}}-E_{b})\\ w(u_{1})=A\exp(-\frac{E_{m}-\alpha_{1}u_{1}}{kT}) \end{array}$$

This approach supposes the introduction of internal state variable *w*, which is determined by the fraction of the insulator region occupied by filaments. The change in this state is associated with the processes of migration of oxygen ions (vacancies) with the height of the effective migration barrier *E_m_*. In turn, the migration is provided by the Joule heating *kT* and applied electric voltage *u*_1_. The total current density *j* through the memristor is the sum of the linear *j_lin_* and nonlinear *j_nonlin_* components. The former corresponds to ohmic conductivity with resistivity *ρ*, whereas the latter is determined by the transport of charge carriers through defects in the regions of the insulator not occupied by filaments (including those in the filament rupture region). It was previously found that, in the insulating state of the studied ZrO_2_-based memristive devices, the current transport is implemented by the Poole–Frenkel mechanism with an effective barrier *E_b_* [62]. The smooth transition between high- and low-resistance states (HRS and LRS, correspondingly) is determined by the dynamic contribution to the total current of the conductive filaments and, therefore, the state variable. In Equation (2), *b*, *α*_1_, and *A* are coefficients derived from experimental data. In our numerical simulations we used the Runge–Kutta integration methods for stochastic differential equations in Matlab [63,64,65].

In order to compare the experimentally observed dynamics of the memristive device with the results of numerical simulations, we needed to take into account stochasticity of microscopic processes leading to a change in the internal state *w* of the dynamical system. Random fluctuations of the normal distribution were added to energy barrier *E_m_* for ion hopping (dispersion 10%), energy barrier *E_b_* for electron jumps in the Poole–Frenkel conduction mechanism in the HRS (dispersion 1%), and ohmic resistance *ρ* of the structure in the LRS (dispersion 10%). This led to the scattering of the experimental current–voltage characteristics. The finite spread of the switching voltages is mainly related to the stochasticity of the energy barrier for ions, whereas the change in the resistive states from cycle to cycle is associated with the electron transport stochasticity.

One-way communication between two neurons through the memristive device was modeled by the following equations:

(3) $$\begin{array}{l} \frac{du_{1}}{dt}=f(u_{1})-v_{1}\\ \frac{dv_{1}}{dt}=\varepsilon_{1}(g(u_{1})-v_{1})-I_{1}\\ \frac{du_{2}}{dt}=f(u_{2})-v_{2}+j(u_{1})Sd\\ \frac{dv_{2}}{dt}=\varepsilon_{2}(g(u_{2})-v_{2})-I_{2} \end{array}$$

where *d* is the equivalent load resistance, *j*(*u*_1_) is the current density through the memristive device, *S* is the area of conductive filaments obtained from the experiment, and *ε* is a small recovery parameter. The signal from the presynaptic neural generator (*u*_1_) was sent to the postsynaptic neural generator (*u*_2_) through the memristive device.

Thus, the two neurogenerators were connected in such a way that part of the current *j*(*u*_1_) generated by the presynaptic neuron passed through the load resistor, which was connected in series with the memristive device, before reaching the postsynaptic neuron. The initial conditions and model parameters corresponded to the experimental conditions. In particular, both neural oscillators were initially in a self-oscillatory regime.

**Figure 1:**
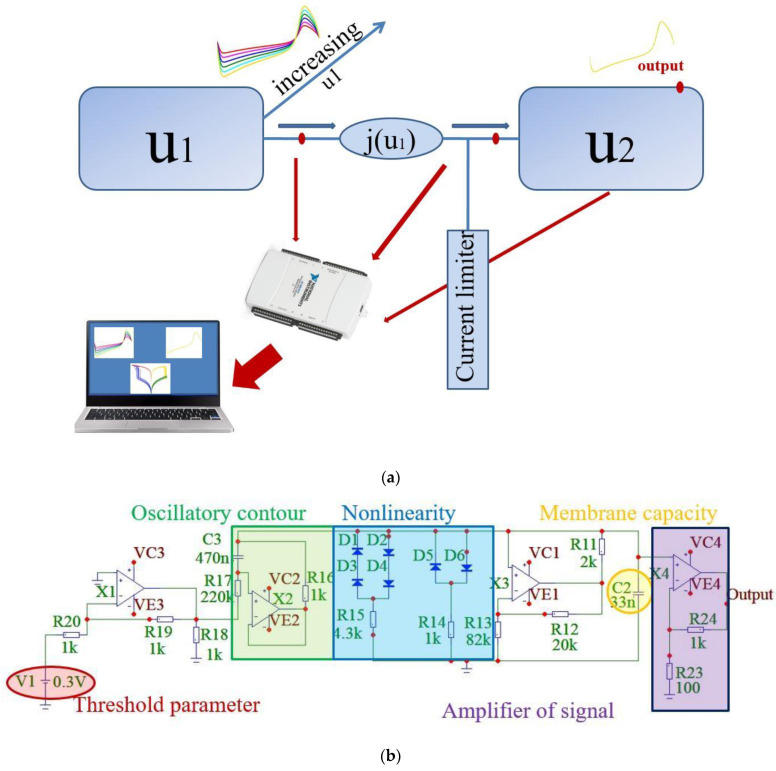
The system description: (**a**) block diagram of the interaction between presynaptic (*u*_1_) and postsynaptic (*u*_2_) electronic neurons through a memristive device. The neurons are initially in an oscillatory regime. The output of the presynaptic neuron is increased during the experiment; (**b**) analog electrical circuit of the FitzHugh–Nagumo neuron. The inductance is implemented by the circuit with operational amplifier, cubic nonlinearity is set using diodes D1–D6, capacitor C2 is related to the capacitance of the neuron membrane, and potential V1 is associated with an equilibrium controlled by the power source.

The designed neuromorphic circuit consisted of an FHN electronic circuit, a memristive device formed by the thin-film metal–oxide–metal nanostructure based on yttria-stabilized zirconia (Au/Zr/ZrO_2_(Y)/TiN/Ti) [66], and a load resistor (Figure 1a). This memristive interface operated as follows. The electronic FHN neuron generated a pulse signal that affects the memristive device and thus modulates the oxidation and recovery of conductive filaments in the oxide film of the memristive device. The analog electronic FHN neuron consisted of the following blocks: an oscillatory contour unit, a nonlinearity unit, and an amplifier unit (see Figure 1b). The detailed design of this device is described in [7,22]. The FHN neural generator demonstrates the main qualitative features of neurodynamics: the presence of an excitability threshold and the existence of resting and spiking regimes. These regimes were controlled using a potentiometer. The spiking frequency was varied in the range of 10–150 Hz, the spike duration in the range of 10–25 ms, and the spike amplitude *u*_1_ in the range of 1–6 V.

In this work, we used the National Instruments USB-6212 data acquisition system, which consists of a digital-to-analog converter (DAC) and two analog-to-digital converters (ADC). The data acquisition system was controlled using LabVIEW software. The pre-recorded neuron-like signal was applied to a memristive device with a sampling frequency of 5 kHz via the DAC. The ADCs recorded the voltage drop across the memristive device and the load resistor, which made it possible to calculate the memristive device resistance in real time. The potential difference across the memristive device (*R_m_*) and the load resistor (*R*_2_) was digitized at a sampling frequency of 10 kHz. Matlab was used to analyze the results.

After testing and tuning, the neuron-like oscillators were connected through the memristive device. Both analog neurogenerators were turned in the oscillatory regime. Under the neuron-like signal action, the memristive device changed its state from high resistive to low resistive. The amplitude of the presynaptic neuron was adjusted by the potentiometer in order to obtain a frequency-locking regime between two oscillators.

## 3. Results and Discussion

**Figure 2:**
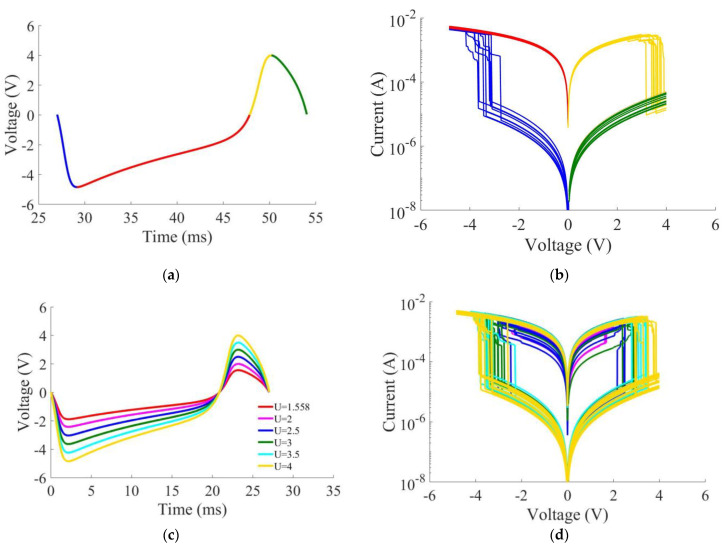
Experimental resistive switching in the response to a neuron-like signal: (**a**) neural-like pulse. The blue, red, yellow, and green colors show, respectively, the curve segments increasing from 0 to −5 V, from −5 to 0 V, from 0 to 4 V, and 4 to 0; (**b**) *I–V* curves. Each colored *I-V* section corresponds to a colored section of the input signal to memristor; (**c**) increasing amplitude of the neuron-like signal. The red, purple, blue, green, light blue, and yellow curve corresponds, respectively, to the peak amplitude of 1.558 V, 2 V, 2.5 V, 3V, 3.5 V, and 4 V; (**d**) resistive switching of the memristive device under the action of corresponding neuron-like signals on the I-V curves. Each colored *I–V* curve corresponds to a colored curve of the input signal to memristor.

The output signal of the presynaptic electronic neuron is shown in Figure 2a. This signal is applied to the memristive device. The used neuron-like signal (*u*_1_) is asymmetric (the minimum voltage is −5 V and the maximum voltage is 4 V) due to the asymmetry of the current–voltage characteristic (*I–V* curves) of the memristive device. For a more detailed study of the effect of the neuron-like signal on the memristive device, the curve in Figure 2a is visually divided into four intervals with different colors. Each interval corresponds to a specific fragment of the *I–V* curves in Figure 2b. The *I–V* curves in Figure 2b display the switching between LRS and HRS. The RESET process (switching from LRS to HRS) occurs with a positive voltage and SET (switching from HRS to LRS) with a negative voltage. The scattering of the *I–V* curves in Figure 2b results from random fluctuations applied to the memristor parameters *E_m_*, *E_b_*, and *ρ*. Figure 2c demonstrates the increase in the amplitude of the presynaptic neuron from 1.558 V to 4 V. Figure 2d shows that, even when exposed to a small amplitude signal of 2 V (purple curve), the memristive device can switch from HRS to LRS.

The laboratory memristor demonstrates different responses to an input signal with a small stochastic spread. Figure 2d shows that, for one curve in Figure 2c, with the yellow curve used as an example, the numerical memristor model yields 10 possible curves (also a yellow color) with a small spread. The *I–V* curves in Figure 2d illustrate the effect of stochastic switching in the memristor response to the voltage signals of the corresponding amplitudes. Since memristor conductivity is adaptively changed according to the input signal, the memristive device demonstrates the property of plasticity.

**Figure 3:**
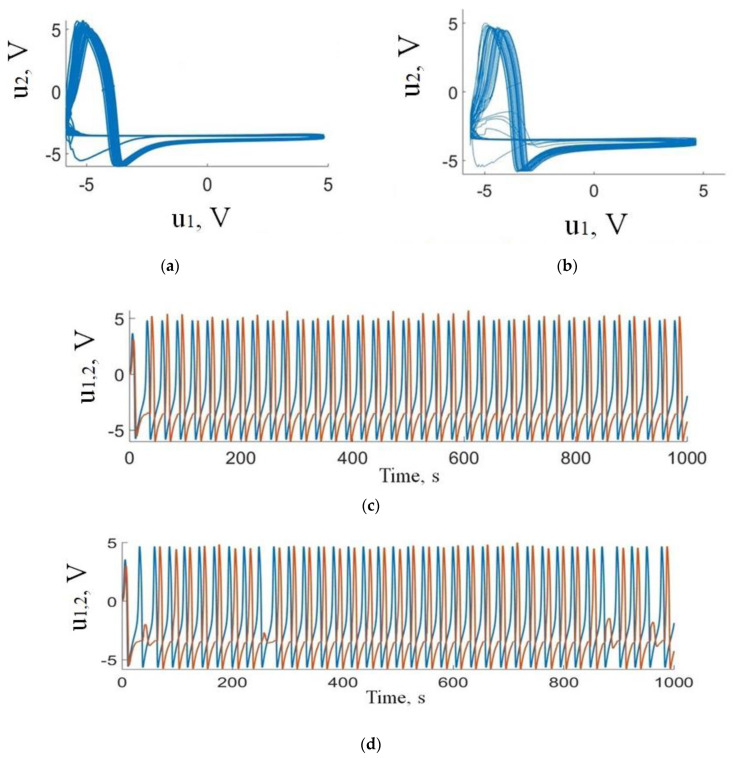
Results of numerical simulations of the dynamics of FHN neuron generators with memristive coupling: (**a**,**b**) phase portraits and (**c**,**d**) time series representing (**a**,**c**) 1:1 and (**b**,**d**) intermittent frequency-locking regimes. Blue and red curves show action potentials of presynaptic (*u*_1_) and postsynaptic (*u*_2_) neurons, respectively.

There is a threshold value of the amplitude (*u*_1_) of the neuron-like signal at which the memristor state switches at each spike. At high amplitudes of the input signal (*u*_1_), the system enters a state of extreme resistance and does not respond to each spike anymore. The memristive device remains in this state. The switching degree strongly depends on the internal changes in the memristive device related to the interrelated transport phenomena in oxide dielectrics, due to electric potential gradients, ion concentration, and local heating [67,68]. These reasons result in the partial recovery and oxidation of conducting filaments in the oxide film. The corresponding dynamical change in conductivity is limited by the applied voltage and leads to the modulation of the strength of neuron coupling and different types of synchronization. In the course of the study, the optimal coupling strength is *z* = *j*(*u*_1_); SR = (0.02–0.06) for 1:1 frequency-locking (Figure 3c) and *z* = (0.06–0.095) for intermittent synchronization (Figure 3d).

**Figure 4:**
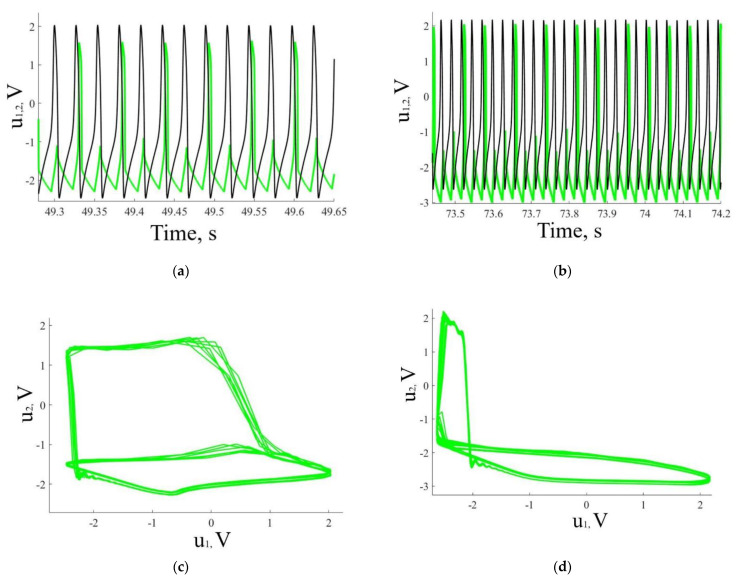
Experimental results demonstrating frequency-locking of FHN electronic neurons coupled by the memristive device: (**a**,**c**) time series and (**b**,**d**) phase portraits representing (**a**,**b**) 2:1 and (**c**,**d**) intermittent frequency-locking regimes. Black and green curves show action potential of presynaptic (*u*_1_) and postsynaptic (*u*_2_) neurons, respectively.

The experiments show that, when the amplitude of the presynaptic neuron *u*_1_ is varied from 1.6 to 2 V, the oscillation frequencies of the coupled neurons are locked either as 2:1 (Figure 4a) or 3:1 (Figure 4b), i.e., the presynaptic neuron *u*_1_ fires the postsynaptic neuron *u*_2_ twice or thrice. This ratio can be randomly changed when chaotic synchronization is reached at higher voltage amplitudes. Although the phase portraits obtained numerically and experimentally do not completely match, the experiment confirms the diversity of phase-locking regimes predicted by the model. Moreover, our model demonstrates dynamics close to the experimentally observed one, despite of to the first-order memristor model, if the stochasticity of switching is accounted for.

The stochasticity is an inalienable property of resistive-switching devices, enabling the so-called stochastic plasticity used to mimic neural synchrony in a simple electronic cognitive system [69]. To the best of our knowledge, the present work is the first attempt to study this important phenomenon both numerically and experimentally. In our case, the stochasticity is modeled through the introduction of fluctuations in the model parameters in a way similar to [70]. Recently, Agudov et al. [71] developed a more generic stochastic model of a memristive device that can be further used to adequately describe the observed complex dynamics of the proposed memristive interface. Another option is to use the deterministic, but at the same time higher-order memristor models based on two or more state variables in order to simulate the experimentally observed intermittency route to chaos [72].

## 4. Conclusions

In this work, we have studied the dynamics of two coupled FitzHugh–Nagumo neuron generators coupled through a memristive device of a metal–oxide type that adapts the synaptic connection according to the amplitude of the presynaptic neuron oscillations. The stochastic switching of the memristive device from a high-resistance state to a low-resistance state is achieved by the variation of the internal parameters. Therefore, the memristive synaptic device demonstrates the property of stochastic plasticity. Different synchronous regimes were observed, including 1:1, 2:1, and 3:1 frequency-locking, intermittent synchronization, and more complex dynamics. Its relative compactness and high sensitivity make the proposed neuromemristive device very promising for biorobotics and other bioengineering applications [73].

## Data Availability

The data that support the findings of this study are available from the corresponding author upon reasonable request.
